# *Anaplastic lymphoma kinase* (*alk*), a neuroblastoma associated gene, is expressed in neural crest domains during embryonic development of *Xenopus*

**DOI:** 10.1016/j.gep.2021.119183

**Published:** 2021-05-19

**Authors:** Marcela M. Moreno, William B. Barrell, Annie Godwin, Matthew Guille, Karen J. Liu

**Affiliations:** 1 Centre for Craniofacial and Regenerative Biology, King’s College London, London SE1 9RT, UK; 2 European Xenopus Resource Centre, School of Biological Sciences, University of Portsmouth, Portsmouth PO1 2DY, UK

**Keywords:** *anaplastic lymphoma kinase*, *alk*, *ltk*, neuroblastoma, neural crest, *Xenopus*

## Abstract

Neuroblastoma is a neural crest-derived paediatric cancer that is the most common and deadly solid extracranial tumour of childhood. It arises when neural crest cells fail to follow their differentiation program to give rise to cells of the sympathoadrenal lineage. These undifferentiated cells can proliferate and migrate, forming tumours mostly found associated with the adrenal glands. Activating mutations in the kinase domain of anaplastic lymphoma kinase (ALK) are linked to high-risk cases, where extensive therapy is ineffective. However, the role of ALK in embryonic development, downstream signal transduction and in metastatic transformation of the neural crest is poorly understood. Here, we demonstrate high conservation of the ALK protein sequences among vertebrates. We then examine *alk* mRNA expression in the frog models *Xenopus laevis* and *Xenopus tropicalis*. Using *in situ* hybridisation of *Xenopus* embryos, we show that *alk* is expressed in neural crest domains throughout development, suggesting a possible role in neuroblastoma initiation. Lastly, RT-qPCR analyses show high levels of *alk* expression at tadpole stages. Collectively, these data may begin to elucidate how *alk* functions in neural crest cells and how its deregulation can result in tumorigenesis.

## 1 Introduction

Anaplastic lymphoma kinase (ALK) is a membrane receptor tyrosine kinase of the insulin receptor superfamily (Allouche, 2007). ALK was first identified in cancers where chromosomal rearrangements caused the fusion of its intracellular domain with other proteins. In patients with anaplastic large cell non-Hodgkin lymphoma, translocation of chromosomes 5 and 2 led to the fusion of the nucleophosmin (*NPM*) and *ALK* genes. This hybrid transcript contains the *NPM* promoter and the ALK catalytic domain, thus leading to an aberrant expression and constitutive tyrosine kinase activity in lymphocytes (Morris et al., 1994). In cases of non-small cell lung cancer, an inversion in chromosome 2p resulted in a fusion of echinoderm microtubule-associated protein-like 4 (EML4) and ALK proteins. This hybrid protein induced tumorigenesis *in vivo* (Soda et al., 2007). Since then, many novel ALK partner proteins have been found and ALK has become a therapeutic target for cancer treatment (Cao et al., 2019). Moreover, amplifications of the *ALK* gene are also a common genetic alteration found in different paediatric cancers. This happens when replication errors cause gene copy number gains, leading to the translation of truncated or full-length ALK protein and, thus, resulting in increased kinase activity (Yael P. Mossé, 2016; Takita, 2017). All in all, the *ALK* gene is a *locus* of genomic instability and plays a pivotal role in tumorigenesis.

ALK is mostly associated with neuroblastoma, the most common and lethal extracranial solid tumour of infancy, causing 15% of childhood cancer-related deaths. The prognoses vary from spontaneous regression to aggressive metastasis in high-risk cases (around 90% of total of cases) (Heck et al., 2009; Johnsen et al., 2019). These tumours derive from neural crest cells. In embryos, neural crest cells are a multipotent stem cell population arising from the neural plate border. They undergo epithelial-mesenchymal transition (EMT), allowing them to migrate to their final destination, where they can differentiate into a myriad of cell types, such as pigmented cells, neurons, cranial bone and cartilage (Green et al., 2015; Theveneau & Mayor, 2012a). Neuroblastoma tumours consist mostly of undifferentiated cells and form in the medulla or paraspinal ganglia of the adrenal glands (Maris, 2010; van Groningen et al., 2017). Thus, it is postulated that they arise from a failure of neural crest cells to follow their differentiation program into the sympathoadrenal lineage (Marshall et al., 2014). How this malignant transformation of neural crest cells occurs remains unknown.

Mutations of *ALK* pose a high susceptibility to neuroblastoma (Maris, 2010). These are usually missense mutations leading to activation of the ALK kinase domain. The most common mutational hotspots are F1174 and R1275 (both within the kinase domain), found in sporadic and familial cases, respectively (Carén et al., 2008; Janoueix-Lerosey et al., 2008; Yaël P. Mossé et al., 2008). These mutations cause ALK activation in a ligand-independent manner and increase treatment resistance (Berry et al., 2012; Montavon et al., 2014). However, the effects of aberrant ALK activation during embryonic development are poorly understood. Therefore, studying the normal function of ALK will contribute to elucidating its role in neuroblastoma initiation.

In physiological conditions, ALK binds to a ligand and undergoes dimerization and activation through trans-autophosphorylation, leading to downstream signalling (Donella-Deana et al., 2005; C. C. Lee et al., 2010). Active ALK serves as a docking site for other kinases and triggers phosphorylation cascades in several interconnected signalling pathways, such as Ras-extracellular signal-regulated kinase (Ras-ERK), Janus kinase 3-signal transducer and activator of transcription 3 (JAK3/STAT3) and phosphatidylinositol 3-kinase (PI3K)-Akt pathway, thus promoting cell survival and proliferation (Chen et al., 2017; H. J. Lee et al., 2014; Lopez-Delisle et al., 2018; Slupianek et al., 2001). However, direct ALK targets are not well known.

Although ALK is a member of the insulin receptor superfamily, it was for some time considered an orphan receptor. This is due to its high substrate specificity, which differs from other insulin receptor superfamily members. More recently, candidate ligands have been identified. Jelly belly (Jeb), a secreted protein responsible for muscle differentiation, was described as an Alk activator in *Drosophila melanogaster* (H. H. Lee et al., 2003). *Drosophila Alk* is expressed in visceral mesoderm and is needed for gut musculature development. Both *Alk* and *jeb* mutants show similar phenotypes of gut malformation and fail to form muscle founder cells (Englund et al., 2003; Wolfstetter et al., 2017). However, Jeb orthologues have not being identified in vertebrates and *Drosophila* Jeb fails to activate mouse ALK (Wang et al., 2019; Yang et al., 2007). More recently, two small secreted proteins, family-with-sequence-similarity-150 A and B (FAM150A and FAM150B) have been shown to bind to human ALK and the closely related leucocyte tyrosine kinase (LTK) (Fadeev et al., 2017; Reshetnyak et al., 2015). In zebrafish, Ltk is more similar to human ALK, and is required for iridophore (neural crest-derived pigmented cells) formation (Mo et al., 2017; Wang et al., 2019). In chicken embryos, Ltk is required for neural crest migration and survival (Vieceli & Bronner, 2018). Like ALK, LTK also signals through the PI3K pathway (Ueno et al., 1997). Also, *LTK* has been shown to be expressed in human leukaemia cells (Maru et al., 1990). However, little is known about the normal function of these kinases during embryonic development.

ALK is likely to play a role in nervous system development. In mice, *ALK* is expressed in the brain and spinal cord of embryos and neonates (Iwahara et al., 1997; Vernersson et al., 2006). More recently, Gonzalez Malagon et al. (2018) showed mRNA and protein expression of ALK in neural crest territories of mouse embryos, such as the branchial arches and neural plate border. Nevertheless, the expression and normal function of Alk has been understudied in other model vertebrates, including *Xenopus*.

*Xenopus* frogs (African clawed frogs) are one of the main animal models for studying developmental biology and are a great model to study embryonic development and human disease (Tandon et al., 2017). *Xenopus* present many experimental advantages, such as synchronised egg laying via hormonal stimulation, which allows easy fertilisation *in vitro*, leading to the generation of hundreds of embryos per fertilisation. Furthermore, these embryos are amenable to genetic manipulation and dissections (Bhattacharya et al., 2015; Horb et al., 2019). *Xenopus laevis* and *Xenopus tropicalis* are the two best-studied species of the genus, with differing advantages. *X. tropicalis* are smaller in size and develop faster, while *X. laevis* embryos are routinely used for embryology and biochemistry (Grainger, 2012). One major difference between the two is their genomics. Due to a genomic duplication, *X. laevis* are allotetraploid, thus they can have up to four copies of the same gene, while *X. tropicalis* are diploid (Harland & Grainger, 2011; Session et al., 2016). All in all, both species are excellent models for studying developmental biology.

The neural crest field has also been advanced greatly due to *Xenopus* studies (Mayor & Aybar, 2001; reviewed in Theveneau & Mayor, 2012b). *Xenopus* embryos allow us to follow key steps of neural crest development: neural plate border induction (stage 13), neural crest specification/delamination (stages 16-18), migration (stage 17 onwards) and differentiation (stage 33 onwards) (Pegoraro & Monsoro-Burq, 2013). Hence, our assessment of *alk* expression in *Xenopus* frogs will be useful for understanding neural crest pathology.

In conclusion, normal ALK function remains elusive. Investigating when and where *alk* is expressed can help elucidate its role in embryogenesis, as well as shed light on how its aberrant activity promotes tumour initiation. Here, we show that *alk* is expressed in neural crest domains at varying levels throughout development of both *X. laevis* and *X. tropicalis* embryos.

## 2 Results

### 2.1 ALK protein is conserved among different model organisms

**Figure 1:**
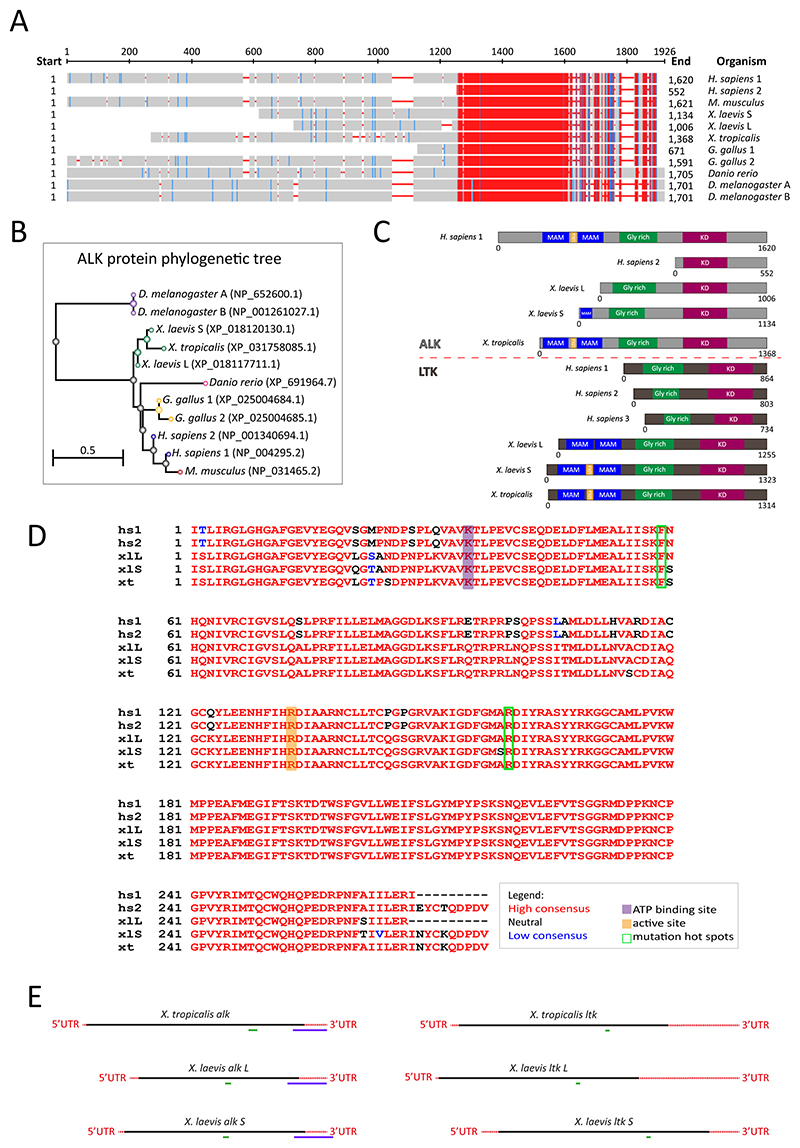
ALK protein is conserved among different organisms. (A) Protein sequence alignment of ALK in different organisms (right). Residue conservation is based on its relative entropy threshold. Red represents highly conserved and blue represents less conserved columns, when there are no gaps. The scale represents the number of amino acids of the consensus alignment. Start and end numbers are the total amino acid count for each sequence. (B) Phylogenetic tree of ALK protein shows similarity between the ALK orthologues (scale bar: 0.5 distance represents 50% difference in sequences) (COBALT). NCBI reference sequence IDs (right). (C) Comparison of ALK and LTK protein domains of *Homo sapiens, Xenopus laevis* and *Xenopus tropicalis* orthologues, highlighting MAM (blue), LDLa (yellow), glycine-rich (green) and kinase domains (purple). (D) Amino acid alignment of human and *Xenopus* ALK kinase domains showing conservation of active site (yellow), ATP binding site (purple) and mutation hotspots (grey arrowheads) (T-Coffee). (E) Schematic of *Xenopus alk* and *ltk* mRNA with UTRs(red) and coding sequences (black) showing RT-qPCR primer amplicons (green) and *in situ* hybridisation antisense probe target (purple) (MAM=meprin, A5 protein, and receptor protein tyrosine phosphatase (MAM) domains; LDLa=low density lipoprotein receptor A domain; Gly-rich=glycine-rich domain; ATP=adenosine triphosphate; UTR= untranslated region).

Using model organisms to study ALK function poses the question of how comparable they are to human ALK and whether the findings are relevant to neuroblastoma. In order to assess how similar the ALK orthologues are, we aligned their protein sequences (Figure 1). To do this, we collected protein sequences of ALK in *Homo sapiens* (isoform 1 and 2), *Mus musculus* (mouse), *X. laevis* (S and L), *X. tropicalis, Gallus gallus* (chicken, isoform 1 and 2), *Danio rerio* (zebrafish) and *Drosophila melanogaster* (isoform A and B). The two *Drosophila* isoforms are identical to each other. Humans have two transcript variants of *ALK*, driven by different promoters, resulting in two isoforms. Human *ALK* isoform 2 has a much shorter N-terminus compared to isoform 1. *Xenopus laevis* L and *Gallus gallus* isoform 1 Alk are also shorter and their functionality remains to be determined. Of note, the sequences for Alk in *Xenopus*, chicken and zebrafish are only predicted and the computational annotation may contain errors.

All sequences were aligned showing an overview of the similarities between them, with nucleotide numbers on the right. These data showed that ALK protein sequences are very similar, particularly in the C-terminal portion, which includes the kinase domain (Figure 1A).

We also visualised the degree of conservation by generating a phylogenetic comparison of ALK protein sequences (Figure 1B). The *Drosophila* Alk sequence differs the most from the other organisms, as expected for a protein from a different subphylum. Among vertebrates, zebrafish Alk is the most divergent sequence, although this may be due to incomplete annotation. Unsurprisingly, among model organisms, the mouse ALK protein is the most similar to human ALK (Figure 1B).

Furthermore, we compared ALK and LTK protein domains of human, *X. laevis* and *X. tropicalis* sequences. To do this, we mapped the meprin, A5 protein (MAM), LDL receptor A (LDLa), glycine-rich and kinase domains within each sequence of both kinases (Figure 1C). MAM domains are thought to function in cell adhesion and mediate homodimerization, but their specific role in ALK or LTK is poorly understood (Beckmann & Bork, 1993; Zondag et al., 1995). The specific functions of the LDLa and glycine-rich domains in ALK are unknown (Palmer et al., 2009; Roskoski, 2013). *D. melanogaster* Alk with mutated glycine residues in its glycine-rich domain is not functional (Lorén et al., 2003a). Human ALK has two MAM domains in its extracellular portion, as does *X. tropicalis* Alk, while *X. laevis* isoforms do not. *X. laevis S* Alk contains one MAM domain and *X. laevis* L Alk does not have it. Interestingly, the *Xenopus* Ltk orthologues contain two MAM domains in their extracellular portion, while the *Homo sapiens* isoforms do not (Figure 1C). We then aligned the kinase domain amino acid sequences of ALK highlighting key residues, such as adenosine triphosphate (ATP) binding site, active site and two of the most common mutation hotspots (F1174 and R1275). Human and *Xenopus* ALK kinase domains showed striking similarities (Figure 1D). ATP binding site, active site and mutation hotspots are the same residues in these sequences. Collectively, these data suggest that ALK protein sequences are similar between humans and model organisms, especially in the kinase domain, which might indicate evolutionary conservation. Notably, ALK and LTK proteins also vary in their extracellular portion, particularly in the MAM domains, across human and *Xenopus* orthologues.

### 2.2 *alk* is expressed in neural crest territories throughout *Xenopus* development

**Figure 2:**
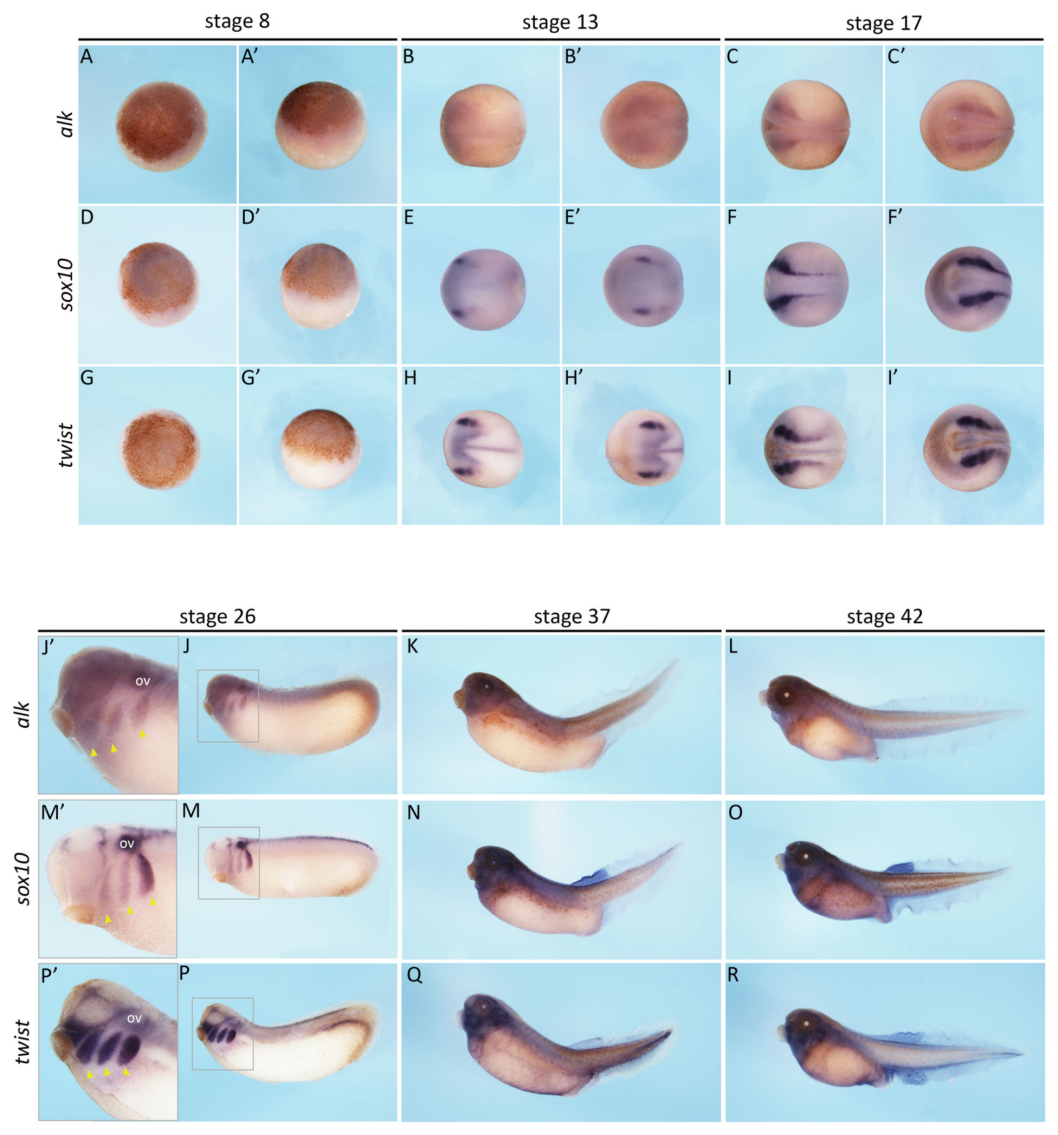
*alk* expression pattern in *Xenopus laevis*. mRNA *in situ* hybridisation for *alk* and neural crest markers, *sox10* and *twist*, in *Xenopus laevis* embryos at stages 8, 13, 17, 26, 37 and 42 (Nieuwkoop & Faber, 1994). Animal (A, D and G) and lateral (A’, D’ and G’) view of stage 8 embryos (*alk* n=11, *sox10* n=5, *twist* n=4). Dorsal (B, E and H) and anterior (B’, E’ and H’) view of stage 13 embryos (*alk* n=12, *sox10* n=4, *twist* n=2). Dorsal (C, F and I) and anterior (C’, F’ and I’) view of stage 17 embryos (*alk* n=7, *sox10* n=4, *twist* n=4). Lateral view (J, M and P) of stage 26 embryos and zoomed insets (grey rectagles) of embryos heads (J’, M’ and P’), showing otic vesicles (OV) and neural crest streams (yellow arrowheads) (*alk* n= 8, *sox10* n=7, *twist* n=5). Lateral view (K, N and Q) of stage 37 embryos (*alk* n= 10, *sox10* n=7, *twist* n=5) and stage 42 embryos. Lateral view (L, O and R) of stage 42 embryos (*alk* n= 11, *sox10* n=5, *twist* n=7).

**Figure 3:**
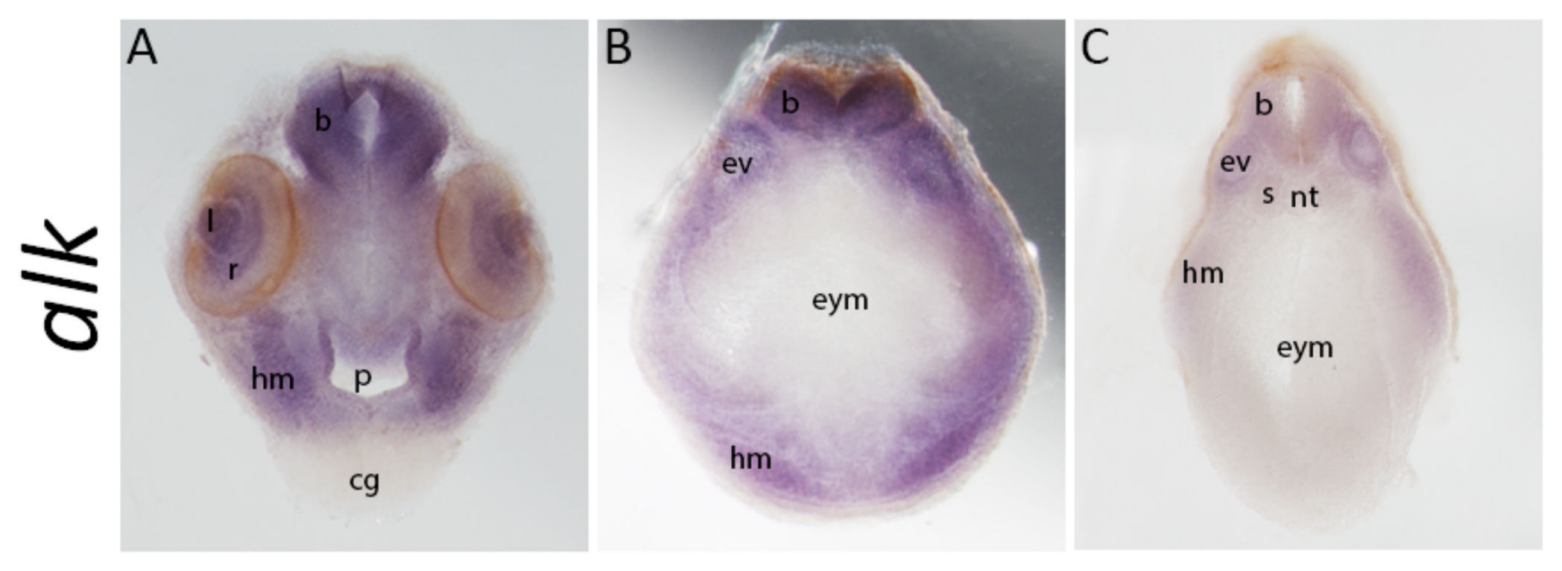
*alk* is expressed in head structures of *Xenopus laevis* tadpoles. Coronal sections of *Xenopus laevis* tadpoles at stage 37, after *in situ* hybridisation, reveal internal structures with *alk* expression (Nieuwkoop & Faber, 1994). Anterior to posterior (A-C) 150 µm sections at the levels of the optic cup (A), ear vesicle (B) and somites (C) (r=retina, l=lens, p=pharynx, b=brain, hm=head mesenchyme, cg=cement gland, nt=notochord, s=somite, ev=ear vesicle, eym=endodermal yolk mass).

**Figure 4:**
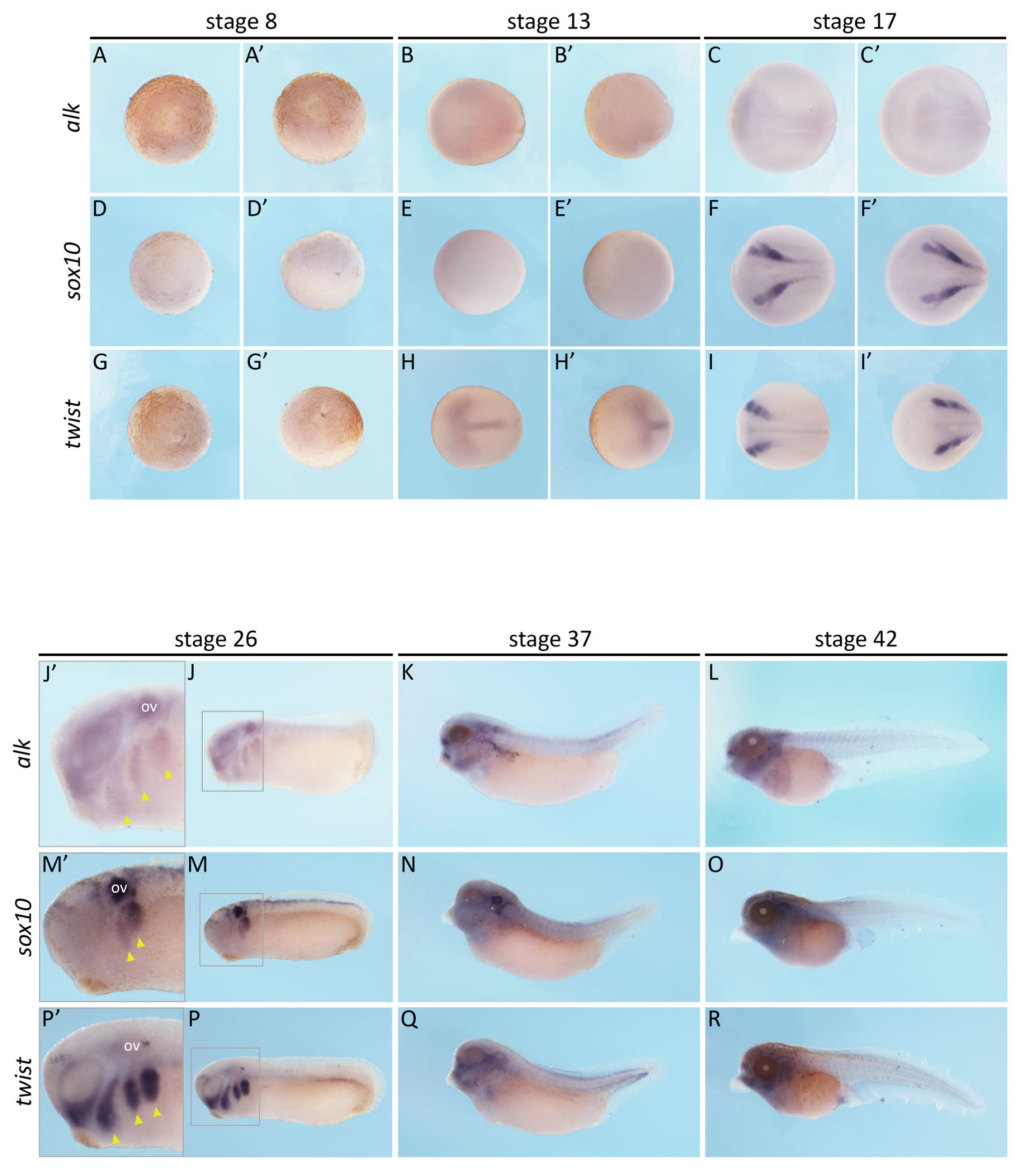
*alk* expression pattern in *Xenopus tropicalis*. mRNA *in situ* hybridisation for *alk* and neural crest markers, *sox10* and *twist*, in *Xenopus tropicalis* embryos at stages 8, 13, 17, 26, 37 and 42 (Nieuwkoop & Faber, 1994). Animal (A, D and G) and lateral (A’, D’ and G’) view of stage 8 embryos (*alk* n=13, *sox10* n=8, *twist* n=7). Dorsal (B, E and H) and anterior (B’, E’ and H’) view of stage 13 embryos (*alk* n=9, *sox10* n=3, *twist* n=5). Dorsal (C, F and I) and anterior (C’, F’ and I’) view of stage 17 embryos (*alk* n=5, *sox10* n=4, *twist* n=4). Lateral view (J, M and P) of stage 26 embryos and zoomed insets (grey rectagles) of embryos heads (J’, M’ and P’), showing otic vesicles (OV) and neural crest streams (yellow arrowheads) (*alk* n= 31, *sox10* n=31, *twist* n=21). Lateral view (K, N and Q) of stage 37 embryos (*alk* n= 6, *sox10* n=5, *twist* n=4) and stage 42 embryos. Lateral view (L, O and R) of stage 42 embryos (*alk* n= 10, *sox10* n=4, *twist* n=4).

Considering the embryonic origin of neuroblastoma, we wondered where *alk* would be expressed during development in *Xenopus* embryos. Moreover, with differences between Alk isoforms in *X. tropicalis* and *X. laevis*, we speculated whether they would present different expression patterns as well (Figure 1C). To do this, we synthesised an mRNA antisense probe to recognise *X. laevis* (both isoforms) and *X. tropicalis alk* transcripts and performed *in situ* hybridisation at different stages of development (Figures 1E and Figures 2-4). Embryos of both species were collected at stages 8, 13, 17, 26, 37 and 42 (Nieuwkoop & Faber, 1994) and compared to expression patterns of neural crest markers, *sox10* and *twist*. These are key transcriptional regulators required during neural crest migration and specification (Honoré et al., 2003; Lander et al., 2013).

It is worth noting that at stage 8, embryos have not undergone mid-blastula transition (when zygotic transcription starts), meaning their transcripts at this point are inherited maternally, in the egg. At this stage, *alk* expression is barely detected in either species (Figures 2A-A’ and 4A-A’). The neural crest markers *sox10* (Figures 2D-D’ and 4D-D’), and *twist* (Figures 2G-G’ and 4G-G’), are also not detected, which is expected. By stages 13 and 17, *alk* expression begins to appear at the neural plate border and neural folds (Figures 2B-C’ and 4B-C’), corresponding to neural crest territories, as seen by *sox10* (Figures 2E-F’ and 4E-F’) and *twist* (Figures 2H-I’ and 4H-I’) expression. At stage 26, *alk* expression is seen in otic vesicles (ov) and neural crest streams (yellow arrowheads) (Figures 2J-J’ and 4J-J’). At tadpole stages 37 and 42, *alk* seems to be expressed more broadly, in the head and dorsal trunk of embryos (Figures 2K-L’ and 4K-L’).

Additionally, we wondered how *alk* is expressed in internal structures in the developing head. To do this, we performed cross-sections through the head of *X. laevis* tadpoles after *in situ* hybridisation for *alk* (Figure 3). We can see that *alk* is expressed in neural ectoderm structures, such as the brain, as well as the lens and retina layers in the eye (Figure 3A). In more posterior sections of the head, we also visualise *alk* expression in the ear vesicles (Figure 3B-C). Furthermore, *alk* expression can be seen in the head mesenchyme across all sections, around the optic cup and pharynx (Figure 3A) and more ventrally around the endodermal yolk mass (Figure 3B). We also observed enriched expression in the epithelia lining the pharynx. Adjacent to the pharynx, we see expression in the mandibular mesenchyme (Figure 3A), notably in a pattern that appears to be neural crest-derived skeletogenic condensations (Kurth et al., 2012; Square et al., 2015). Taken together, *alk* expression is distributed in different structures of the head of *X. laevis* tadpoles, such as brain, optic cup, ear vesicle and head mesenchyme.

Both *X. laevis* and *X. tropicalis* showed similar *alk* expression patterns. *X. tropicalis* embryos had weaker staining (Figures 2A-C, 2J-L and 4A-C, 4J-L), but this was also consistent with neural crest domains, as seen when compared to *sox10* (Figures 2D-F, 2M-O and 4D-F, 4M-O) and *twist* (Figures 2G-I, 2P-R and 4G-I, 4P-R) expression. Sections of the head of *X. laevis* tadpoles also revealed *alk* expression in neural ectoderm derivatives and head mesenchyme. These data suggest that *alk* is expressed in (but not limited to) the neural crest throughout *Xenopus* embryonic development.

### 2.3 *alk* and *ltk* expression levels vary in different *Xenopus* embryos stages

**Figure 5:**
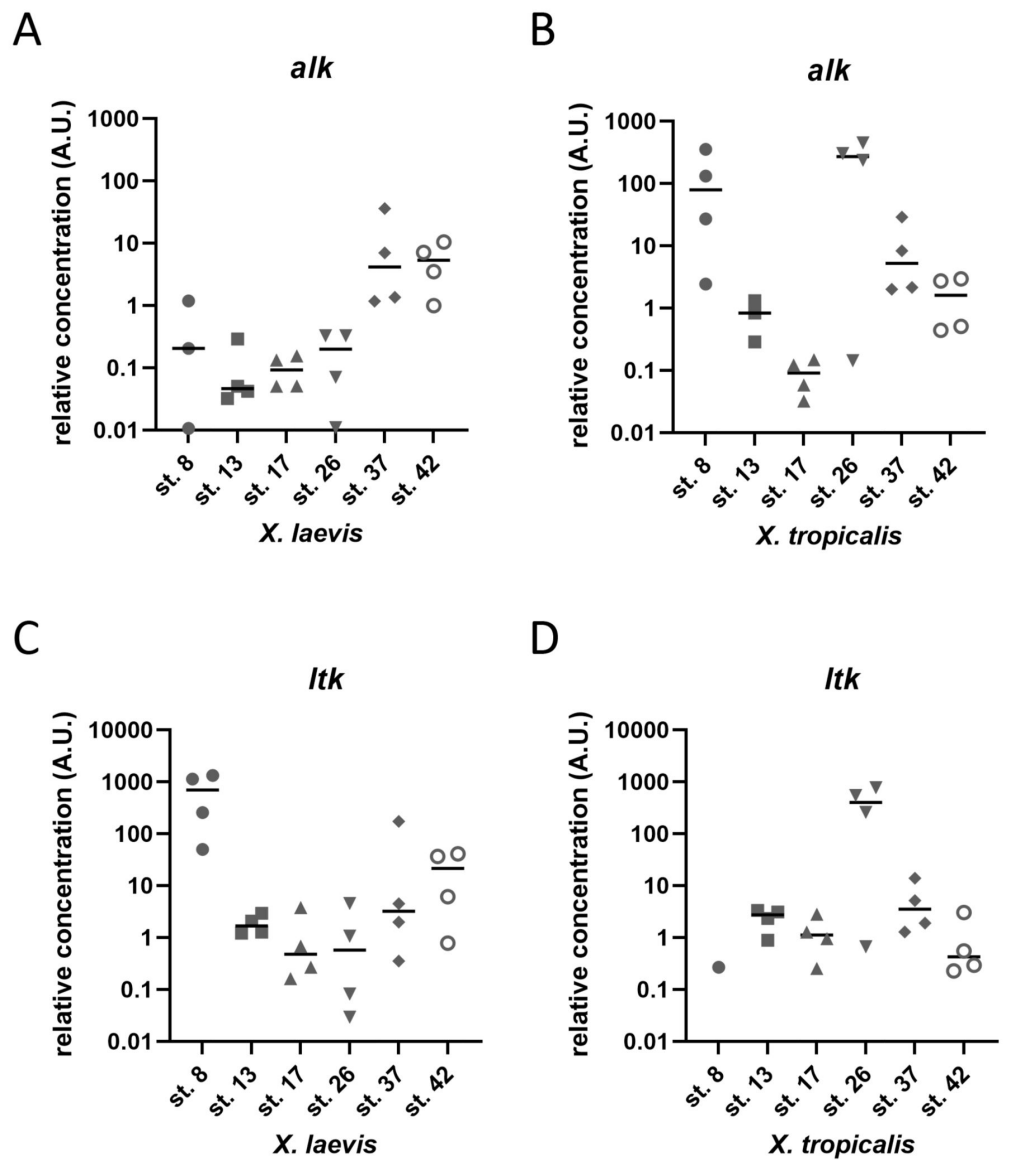
Expression of *alk* and *ltk* in *Xenopus laevis* and *Xenopus tropicalis*. RT-qPCR of embryos samples at stages 8, 13, 17, 26, 37 and 42 (Nieuwkoop & Faber, 1994). Graphs show the absolute quantification (in Log10 scale) of *alk* (A and B) and *ltk* (C and D) levels, relative to the expression of the housekeeping gene, *ef1α. Xenopus laevis* (A and C) and *Xenopus tropicalis* (B and D) embryos were pooled in sets of 3 embryos for each stage and used for RNA extration, cDNA synthesis and RT-qPCR. Each icon represents one replicate (pool of 3 embryos). When there are less than 4 icons, no product was detected for these replicates. Bars represent the arithmetic mean between replicates of each stage. (A.U.=arbitrary units).

Next, in order to get a quantitative analysis of *alk* expression during *Xenopus* development, we performed RT-qPCRs. Primers were designed to specifically target *Xenopus alk* (both isoforms of *X. laevis* and *X. tropicalis* isoform) (Figure 1E and table 1). Embryos at stages 8, 13, 17, 26, 37 and 42 of *X. laevis* and *X. tropicalis* were collected and processed for cDNA synthesis and subsequent RT-qPCR (Nieuwkoop & Faber, 1994). To our surprise, the two species showed quite different trends in *alk* expression. In *X. laevis, alk* shows increasing expression levels from stages 13 to 42 (Figure 5A). On the other hand, in *X. tropicalis, alk* expression levels fluctuate, decreasing from stage 8 to stage 17, showing a high peak at stage 26, and decreasing at stages 37 and 42 (Figure 5B). Stage 26 differences were the most surprising, as the expression domains seem to be similar in the *in situ* hybridisations; of course these cannot be quantitatively compared as in the RT-qPCR data (Figures 2J, 4J and 5A-B).

We also assessed expression levels of *ltk*, since it is closely related to *alk* and their functions could overlap (Figure 1C). However, *ltk* expression levels did not vary as much as *alk* across different stages of development of both *X. laevis* and *X. tropicalis*, with higher levels at stage 8 and 26 in each respectively (Figure 5C-D). In contrast to the *in situ* hybridisation data, which is less sensitive (and not quantitative), *alk* expression can be seen by RT-qPCR at stage 8, as for *ltk*. This suggests both kinases are likely to be maternally expressed.

Taking into account experimental variability, both species present low *alk* expression levels at stage 17, when neural crest cells are induced, and higher levels at tadpole stages 37 and 42, when neural crest cells begin differentiating. Conversely, *ltk* expression did not vary as much. However, *alk* and *ltk* were differently expressed in *X. laevis* and *X.tropicalis* when compared stage by stage, which might reflect their genomic distinctions.

## 3 Discussion

Here we show a previously unknown developmental expression pattern of *alk* in *X. laevis* and *X. tropicalis* by *in situ* hybridisation and qPCR. These data provide spatial and quantitative analyses of *alk* expression throughout embryonic development. Previous studies have reported *alk* expression in the developing nervous system (Iwahara et al., 1997; Vernersson et al., 2006). However, data on earlier stages of development were still missing. Our work reveals *alk* expression as early as stage 13, as seen by *in situ* hybridisation, and detected at stage 8 via RT-qPCR (Figures 2-5), which is sustained until tadpole stages (Nieuwkoop & Faber, 1994). Sectioning of tadpoles revealed *alk* expression in the head mesenchyme, a neural crest domain, as well as in ectodermal derivatives (Figure 3). These data corroborate the hypothesis of Alk function playing a role in the developing nervous system, particularly in early neural crest cells. As neuroblastoma consists of mostly undifferentiated cells, a disruption of ALK activity occurring in precursors of the sympathoadrenal lineage might be triggering tumorigenesis. In fact, it has been shown that patient-derived neuroblastoma cell lines with more mesenchymal RNA profiles clustered closer to neural crest cell lines than the ones that expressed more adrenergic genes (van Groningen et al., 2017). Furthermore, our RT-qPCR data support the trends seen in RNAseq data available in *Xenopus*, including maternal expression of *alk*, followed by lower levels at neurula stages and an increase towards tadpole stages (Figure 5A-B) (Owens et al., 2016; Session et al., 2016). Conversely, one of the few Alk ligands identified, *fam150a* is thought to be a zygotic gene (data not shown). In physiological conditions, activation of wild-type Alk is thought to be only in a ligand-dependent manner. Thus, investigating the expression pattern of Alk ligands would be crucial for elucidating its activity throughout embryonic development.

Although ALK protein has a well-conserved intracellular portion, especially its kinase domain, its extracellular portion differs among different orthologues (Figure 1A). *X. tropicalis* Alk has two MAM domain, as does human ALK, while *X. laevis* S Alk only has one. These domains are thought to function in cell-cell interactions (Lorén et al., 2003b). Hence, we asked ourselves if the two species would have different expression patterns for the two orthologues. Visually, *X. laevis* and *X. tropicalis* had no remarkable differences in *alk* expression territories throughout embryonic development (Figures 2-4). Conversely, *alk* expression levels quantified by RT-qPCR showed divergences between the two species, particularly at stage 26, when it reaches high levels in *X. tropicalis* and low levels in *X. laevis* (Figure 5 A-B). It is possible that, at this point, the allotetraploid *X. laevis* has sufficient kinase activity, while the diploid *X. tropicalis* has not. Whether the two Alk orthologues function differently remains to be determined.

Finally, *ltk* has been reported to be expressed in neural crest in zebrafish and chicken embryos, but its function in mammals is not well known (Lopes et al., 2008; Vieceli & Bronner, 2018). However, LTK seems to signal thorough some of the same downstream pathways as ALK, such as PI3K, ERK and JAK/STAT. Moreover, *LTK* is expressed in human leukaemia cells and activating mutations (mimicking ALK neuroblastoma-related mutations) were able to induce transformation in haematopoietic cells (Roll & Reuther, 2012; Ueno et al., 1997). Our comparison of LTK protein domains in human and *Xenopus* orthologues shows that only the latter contain MAM domains, different from what we see in ALK (Figure 1C). Thus, it raises the possibility of these two kinases having overlapping functions. Indeed, it has been postulated that they result from a gene duplication occurred during evolution of jawed vertebrates (Wang et al., 2019). Therefore, we assayed *ltk* expression levels in *Xenopus* throughout development. We saw less variable *ltk* expression levels across different stages. It is possible that *ltk* expression is not as developmentally critical as *alk* for neural crest cells. Further studies are needed to elucidate this hypothesis.

## 4 Conclusion

This present study reveals the expression pattern of *alk* in *X. laevis* and *X. tropicalis* embryos. Both species show *alk* expression in neural crest territories. These data will contribute to the understanding of Alk function during embryonic development and neuroblastoma initiation.

## 5 Experimental procedures

### 5.1 Sequence alignments

ALK and LTK protein and mRNA sequences were obtained from the National Center for Biotechnology Information (NCBI) database. Alignments and phylogenetic tree visualisation were done using Constraint-based Multiple Alignment Tool (COBALT). Conservation colouring was selected to highlight conserved residues based on their relative entropy threshold. Sequences in FASTA mode were mapped in GenomeJP motif search, using the Pfam database (Bateman et al., 2002), to identify protein domains and key residues. Kinase domain sequences were aligned using T-Coffee and amino acids were coloured according to consensus value, with 90% for high consensus and 50% for low consensus.

### 5.2 Animal procedures

*Xenopus* embryos were obtained using standard protocols of hormone-stimulation and *in vitro* fertilisation as previously described (Sive et al., 2000). *Xenopus laevis* embryos were cultured in a 1:3 diluted solution of Modified Frog Ringers (MR) (0.1 M NaCl, 1.8 mM KCl, 2 mM CaCl_2_, 1 mM MgCl_2_, 5 mM HEPES-NaOH pH 7.6) at 17°C until reaching desired stages, according to Nieuwkoop & Faber, 1994. *Xenopus tropicalis* embryos were cultured in 0.06X MR at 23°C. Images were taken at a regular light microscope. Animal procedures were performed in accordance with UK Home Office Project Licence P8D5E2773 and PPL70/8983.

### 5.3 Antisense mRNA probe synthesis, *in situ* hybridisation and vibratome sectioning

Whole-mount *in situ* hybridisation was performed according to Sive et al. (2000). Collection of embryos was done at the indicated stages (according to Nieuwkoop & Faber, 1994). They were fixed in 1x MEMFA (100 mM MOPS pH 7.4, 2 mM EGTA, 1 mM MgSO_4_, 3.7% (v/v) formaldehyde) for 2 hours at room temperature, dehydrated in methanol and stored at -4°C overnight. Plasmids containing *sox10, twist* (as per Gonzalez Malagon et al., 2018) and *alk* (*X. tropicalis* EST clone IMAGp998K1516782Q, Source Bioscience) were linearized using restriction enzymes EcoRI (R6011, Promega), for *sox10* and *twist*, and SalI (R6051, Promega), for *alk* and the reaction was incubated at 37°C for one hour. Labelled antisense mRNA probes were synthesized with T3 (P1430, Promega) or T7 (P1440, Promega) polymerases for *sox10* and *twist*, respectively, using Digoxigenin RNA labelling mix (11277073910, Roche). Probes were purified with ProbeQuant G-50 Micro Columns (GE28-9034-08, Sigma). Anti-Digoxigenin-AP, Fab fragments (11093274910, Roche) antibody conjugated with alkaline phosphatase was used to recognize labelled probes at 1:10000 dilution. BM-Purple (11442074001, Roche) was used as a chromogenic substrate. A subset of embryos was embedded in gelatine for vibratome sectioning. They were put in a 20% gelatine from bovine solution (G9382, Sigma) at 55°C for 1 hour. Then, the embryos in gelatine were poured into moulds, positioned to allow coronal sections, and incubated at 4°C until solidified. The blocks were fixed in 4% paraformaldehyde for 3 to 4 days prior to sectioning. The vibratome (VT1000S, Leica) was set to cut 150µm sections.

### 5.4 cDNA synthesis and RT-qPCR

Sets of 4 embryos of each stage were lysed with TRIzol reagent (15596026, Invitrogen) and phase separation and RNA extraction was performed as per Rio et al. (2010). 2 µg of RNA from each pool of embryos were used for cDNA was synthesis with M-MLV reverse transcriptase (M1701, Promega) as per manufacturer. cDNA from samples of stages 37 and 42 were diluted 5 fold and used for top standard (10000x) and series diluted for other standards (5000x, 1000x, 500x, 100x, 10x, 1x). For the RT-qPCR reaction, 1:20 dilution of *X. laevis* and 1:10 dilution of *X. tropicalis* cDNA libraries were used and reaction mix SensiMix SYBR & Fluorescein Kit (QT615-05, Bioline). We performed the RT-qPCR for *alk, ltk* and *ef1α*, as a reference gene. Primer sequences and annealing temperatures are in table 1. The reaction was performed in LightCycler®480 qPCR machine (Roche) and absolute quantification was obtained with their software. Graphs plotted the relative concentration each gene (in Log10 scale), normalised with *ef1α*, with each icon representing a pool of 3 embryos.

## Figures and Tables

**Table 1:** List of RT-qPCR primers

Gene	Sequence	Annealing temperature
*X. laevis alk*	Forward: GGTGACCTCAAGGAAGTGCC Reverse: AGGGTCATTTGCAGAACCCA	60°C
*X. tropicalis alk*	Forward: AACCAGCCTTCCTCCATCAC Reverse: ATCTCCAATCTTGGCCACCC	58 °C
*X. laevis* and *X. tropicalis ltk*	Forward: GTTTTGCAGGGAAGGGAGCC Reverse: CCAGGGCTCTAAGCAGAGTA	58 °C
*X. laevis* and *X. tropicalis ef1α*	Forward: GGATCTGGCAGCGGAACTAC Reverse: GGGGCATATCCAGCACCAAT	58 °C
